# Addressing conflicts of interest in Public Private Partnerships

**DOI:** 10.1186/1472-698X-10-19

**Published:** 2010-07-08

**Authors:** Emmanuel B Omobowale, Michael Kuziw, Melinda Treurnicht Naylor, Abdallah S Daar, Peter A Singer

**Affiliations:** 1 McLaughlin-Rotman Centre for Global Health, University Health Network and University of Toronto, 101 College Street, Suite 406, Toronto, Ontario, Canada

## Abstract

**Background:**

Many articles have been written on conflicts of interests (COIs) in fields such as medicine, business, politics, public service and education. With the growing abundance of Public Private Partnerships (PPPs), often involving complex relationships among the partners, it is important to understand how COIs can be mitigated and managed in PPPs.

**Discussion:**

We wanted to study PPPs, particularly in the areas of global health and agriculture, but discovered no single source of information available to identify and compare various approaches for avoiding and managing COIs in PPPs. This is a significant gap, especially for those wishing to study, compare and strengthen existing COI policies related to PPPs. In order to bridge this gap, we reviewed how PPPs currently address COIs and highlight what might be considered good practice in developing COI policies. We reviewed the online COI policies of 10 PPPs in global health and agriculture, and interviewed two global health PPP chief executives.

**Summary:**

Based on our review of policies and interviews, we conclude that there exists a range of good practices including attention to accountability and governance, acknowledgement and disclosure, abstention and withdrawal, reporting and transparency, and independent monitoring. There appears to be a need for PPPs to interact closely and learn from each other on these parameters and to also place more emphasis on independent external monitoring of COIs as a means of strengthening their major social objectives on which their activities are largely predicated. We also recommend the establishment of a web based database, which would serve as a forum to discuss COI issues and how they can be resolved.

## Background

Conflicts of interest (COIs) have been defined as involving a "set of conditions in which professional judgments concerning a primary interest (such as a patient's welfare or validity of research) tends to be unduly influenced by a secondary interest (such as financial gain)" [1]. COIs can pertain to individuals or entire institutions, and can be financial or non-financial. Much has been written on COIs in fields such as medicine, business, politics, public service, and education. However, little if any guidance has been provided on the methods for managing COIs within the Public Private Partnerships (PPPs) operating in global health and agriculture. Since partnerships between public (by which we mean non profit) and private (by which we mean for profit) organizations will invariably raise issues of COI, the recognition and management of these COIs is critical to the existence of these partnerships.

There are many PPPs operating in health and agriculture. Product Development PPPs (PD-PPPs) like the Medicines for Malaria Venture or the TB Alliance, assist in the development of novel health products to tackle diseases in the developing world. Access PPPs, such as The Global Fund for AIDS, Tuberculosis and Malaria (GFATM) or the Global Alliance for Vaccines and Immunization (GAVI), have the goal of improving access to treatments by providing funding mechanisms for purchase of existing drugs or vaccines. Similarly, PPPs, which operate in the field of agriculture like the Maize and Wheat Improvement Centre (CIMMYT), the International Institute of Tropical Agriculture (IITA), World Agroforestry Centre (ICRAF), African Agricultural Technology (AATF), International Potato Centre (CIP) and Bioversity International (BI) work to develop, through research, novel seedlings and agricultural practices to alleviate hunger and improve farming practices in different parts of the world. The dozens of PPPs working in these fields include "small single product collaborators with industry to large entities hosted in UN agencies of private not-for-profit organizations" [2-5].

Usually, these partnerships are created because the private and public sector partners realize their inability to single-handedly solve the multifaceted problems confronting society. However, there is also the notion that private companies, especially in the healthcare sector, where many PD-PPPs and Access PPPs operate, participate in PPPs, in order to seek "future profits or markets or to control the agenda of international agencies" [6,7]. With billions of dollars flowing through PPPs aiming to improve the health of the global poor or to alleviate hunger through the development of new farming techniques, there is a lot at stake if these primary interests were to be undermined or even seen to be undermined. This is especially true since the trust existing among the partners in a multi-stakeholder organization is a key component of its activities, which enables it to function. The existence of trust among the partners helps to accentuate their sense of commitment to the achievement of the aims and objectives of the PPP and this also helps to facilitate the effective implementation of any COI policy within the organization.

To examine how PPPs address COIs, we reviewed the websites of PPPs which are active in global health and agriculture to describe the suggested practices and guidance contained in their COI policies and in some cases requested these policies (see Table 1). We also examined their websites and interviewed the chief executives of the Human Hookworm Vaccine Initiative of the Sabin Vaccine Institute (Sabin-HHVI) and PATH. Our objective was to understand in depth how PPPs currently address COIs. Given the heterogeneity, this was not a comprehensive survey of every PPP, but rather an attempt to highlight actual practices.

**Table 1:** An Overview of Conflict of Interest Policies of Selected Procurement and PD-PPPs

Publisher	Date of Publication	Conflict of Interest Definition	Conflict of Interest (COI) Guidelines	External Monitoring *(from a source outside the PPP)*	Website
**PUBLIC-PRIVATE PARTNERSHIPS**					

**PATH**	12/2005	The conflict between the private interests and official responsibilities of an *individual *or an *institution *in a position of trust, which may compromise impartiality or integrity or lead to unfair competitive advantage	**Guiding Principles for Managing COI** Values 1. Disclosure 2. Transparency 3. Respect 4. Support 5. Options Responsibilities 1. Acknowledgement 2. Accountability: individual COI 1. Accountability: institutional COI Actions 1. Training staff 2. External monitoring 3. Internal monitoring	No	http://www.path.org (general) http://www.path.org/files/ER_gp_conflict.pdf (COI specific)

**Global Alliance for Vaccines and Immunization** **(GAVI)**	03/2006	A conflict of interest arises whenever the personal or professional interests of an employee or board member are potentially at odds with the best interests of the organization.	**The GAVI secretariat hires Procurement Expert(s) to:** 1. Address COI issues as related to procurement 2. Link up with broader policy on COI	Yes, on some issues procurement experts provided.	http://www.gavialliance.org/resources/jec_23mar2006_AF2.ProcPrince.pdf

**GAVI**	06/2000	See above	GAVI applies specific conflict of interest policies to its different partners. For example, WHO/UNICEF are requested to complete a 'conflict of interest form' when executing contracts and participating in meetings.	See above	http://www.gavialliance.org/resources/oslo_annex2.doc

**The Global Fund**	08/2005	A conflict of interest arises when a Covered Individual participates personally or substantially in an official capacity in any particular matter in which, to his or her knowledge, he or she or an Associated Person or Institution has a financial interest, if the particular matter will have a direct and predictable effect on that interest.	**Transparency and Disclosure** - all covered individuals have a duty to disclose the existence of any conflict of interest and the nature of such conflict - all covered individuals must complete and submit the "Declaration of Interest" to the Secretariat - disclosure statements shall be updated annually and whenever there is a material change in the information they contain - all conflict of interest are immediately disclosed to a Ethics and Conflict of Interest Committee	No	http://www.theglobalfund.org/documents/policies/PolicyonEthicsandConflictofInterestforGlobalFundInstitutions.pdf

**International Maize and Wheat Improvement Centre (CIMMYT)**	02/2008	N/A	**Operational Guidelines for Assessing the Impact of Agricultural Research on Livelihoods** "A conflict of interest should be dealt with openly and transparently so as not to compromise the reliability and credibility of the process and the IA results"	No	http://www.aec.msu.edu/fs2/zambia/sweet/CIMMYT_impact_assessment_guidelines_Rovere-Dixon_2007.pdf (CIMMYT COI policy) http://www.cgiar.org/impact/research/maize.html (CGIAR information on Maize) http://dtma.cimmyt.org/ (Drought Tolerant Maize for Africa Project)

**IITA**	05/1995	All matters which may tend to interfere with the Member's ability to participate in the activities of the Governing Board in a disinterested manner.	**Policy** 1. During consideration of any proposed commitment of funds of IITA to a beneficiary, any trustee who is formally connected with any interested party shall indicate that connection and withdraw from all related activities 2. No member of the board, other than the director general, shall enter into a direct contract with IITA **Implementation** - letters will be sent to individuals considered for election to membership to advice them of the policy so that they may consider whether they are prepared to undertake the obligations required.	No	IITA is a CGIAR research centre that has done extensive work on maize including developing a "Drought Tolerant Maize for Africa" project with CIMMYT. http://www.iita.org/cms/details/maize_project_details.aspx?zoneid=63&articleid=273 (IITA work on maize) http://dtma.cimmyt.org/ (Drought Tolerant Maize for Africa Project) http://www.iita.org/cms/articlefiles/306-conflict.pdf (COI Policy)

**World Agroforestry Centre (ICRAF)**	03/2004	N/A	The policy only mentions conflict of interest once as ICRAF seems to place minimal emphasis on conflict of interest within research.	No	http://www.worldagroforestry.org/downloads/policies%20and%20guidelines/ICRAF_policy_research_ethics.pdf

**African Agricultural Technology Foundation (AATF)**	11/2006	N/A	**"Operational risks** 2. Conflicts of interest at Board or partner level: Board members will be appointed in their personal capacity and not as representative of their institution. AATF Management shall periodically conduct partnership analysis and take appropriate action when cases of conflict of interest are identified."	Yes	http://www.aatf-africa.org/UserFiles/File/strategicdirection1.pdf (AATF Strategy document)

**AATF**	2008	N/A	"These [public-private] partnerships are usually governed by agreements that attempt to identify and articulate shared risks and are viewed as separate from stakeholders"	Yes	http://www.aatf-africa.org/partnerships

**Consultative Group on International Agricultural Research (CGIAR)**		CGIAR provides a legal and symbolic role and a statement to set and reinforce ethical standards, values, policies and basic responsibilities of individual board members	Some examples of the mandate: - To maintain organizational integrity and guarantee that the actions of the Boards are in the best interests of the Center, Board Members need to understand their collective and individual responsibilities to avoid conflict of interest - Ensure that the Center has in place policies needed for effective performance (including conflict of interest) -Individuals should avoid conflict of interest, declare potential conflicts ahead of meetings, and recuse himself or herself from Board debates and decisions on matters for which he or she has a conflict CGIAR's policy is targeted to board members. Guidelines for individual employees or the institution's values itself could not be found. The annex at the back of the document includes a sample self-assessment checklist for CGIAR Boards and Board Members.	No	http://www.cgiar.org/

**Convention of Biological Diversity**	2006	The policy on the webpage (or in hardcopy) is much more extensive than the overview of the guidelines given here.	This PPP's COI policy states that "it would be almost impossible to foresee the different circumstances under which conflict of interest could possibly arise, and that there was limited experience of compliance mechanisms under other international instruments in elaborating what constitutes conflict of interest, or in handling practical cases of conflicts of interest. The rational given for this position is that there is already a procedure of compliance which requires members to serve objectively and in a personal capacity, which taken together with rules of procedure provide general guidelines to conflict of interest.	No	http://www.cbd.Int

**International Potato Centre (CIP)**	N/A	CIP has an external review system comprised of two parts. Center Commissioned External Reviews (CCERS) which originate with the board of trustees and Internally Commissioned External Reviews (ICERS) which originate from management.	The reasons for commissioning a review can be many including: "The desire of the Board or management to have an external opinion of the status, relevance, quality or direction of a particular set of activities. Interestingly, the selection of any board members who want to participate in the reviews must take into account possible conflicts of interest they may have with the reviewing process or subject matter	Yes	http://www.cipotato.org/

**Bioversity International**	N/A	Bioversity International has a whistle-blower policy which protects individuals who whistle-blow, provides guidance for how and where to report instances of wrongdoing including conflict of interest.	The policy is unique in that it protects individuals who report other employees' conflicts of interest. Most policies only cover the obligation of employees to report and manage risks of possible conflict of interests in their own actions, rather than that of their fellow employees. The Whistle-Blower Policy provides the purpose, definition and applicability of the policy. It provides guidance to individual employees on what and when to report, what reporting channels are available, and their rights for feedback and protection.	No	http://www.bioversityinternational.org

These findings will be of interest to the public and private stakeholders who are involved in PPPs in global health and agriculture, as well as the broader global health and agriculture communities who rely on these organizations for novel products and improved access.

## Discussion

### Accountability and Governance

Accountability is at the core of every conflict of interest. Dennis Thompson, who has written extensively on conflicts of interest, argues that accountability encompasses reliance on the good character and by extension, good judgment of the decision marker(s), regulation by a professional body, regulation by government(s) through legislation, and finally, a disclosure of conflicting interests. To ensure accountability, PPPs have boards that supervise their activities. Board members are expected to be individuals who epitomize integrity and who would put the aims and objectives of the organization uppermost in their minds when making decisions, which could affect the success of the PPP. This standard of decision making is expected even when there are other competing interests, which may influence or affect the decision-making progress of the organization.

For example, Sabin-HHVI is a health PD-PPP based at the Sabin Vaccine Institute (SABIN). SABIN is affiliated with the Department of Microbiology, Immunology, and Tropical Medicine of George Washington University (GWU) and uses the laboratories there to conduct research into the development of vaccines for hookworm and other neglected tropic diseases. The President of SABIN-HHVI is also the chairperson of the Department of Microbiology, Immunology, and Tropic Medicine at GWU. During our discussion with the President of SABIN-HHVI, we learned that to prevent any potential COI, two meetings are held annually between representatives of SABIN and GWU in order to review potential or perceived conflicts, and in addition SABI-HHVI employs the services of an attorney from their board to help manage COI issues which might arise.

### Acknowledgement and Disclosure of Possibility of Conflict of Interest

The acknowledgement of the possibility of conflicts of interest was present in all policies we reviewed. These policies are available on the websites of the PPPs. The CEOs of HHVI and GAVI, whom we interviewed, both agreed that as multi-stakeholder organizations, conflicts of interest within PPPs are sometimes inevitable and this should be acknowledged. We also discovered that PPPs must not only be alert to actual conflicts of interest but also to perceived ones, since these too can undermine trust amongst the stakeholders in a PPP.

We can illustrate this point with the example of PATH, an international nonprofit organization dedicated to the development of appropriate health technologies and which is closely associated with multiple PD-PPPs with over 60 companies. During our review of PATH's COI policy [8], we found out that an annual disclosure of COI is mandatory for board members and employees in Finance, Legal Affairs, Procurement and program staff in a position to meaningfully influence the structure or details of any particular public-private partnership. These employees are also required to fill out disclosure forms annually in a bid to identify and address individual conflict of interests. For instance, through this procedure, PATH employees who own stock in companies where PATH is involved in a business or financial relationship are expected to disclose such holdings.

The PATH "Guiding Principles" document provides definitions for concepts such as bias and conflict of interest, and discusses issues of actual and perceived conflicts of interest, individual and institutional conflicts of interest, as well as the direct and predictable effects of conflicts of interest. The "Guiding Principles" document for managing COIs is divided into three parts, namely, "our values", "our responsibilities" and "our actions". In the second section, "our responsibilities", PATH acknowledges that there might be occasions when some measure of bias, inherent in its activities as a non-profit organization, might precipitate individual or institutional conflicts of interest. When such situations occur, the guiding principles stipulate immediate individual or institutional acknowledgement. The concepts of "Disclosure" as well as "Reporting and Transparency", which are also discussed below, are closely aligned with "Acknowledgement of Possibility of Conflict of Interest".

For a PD-PPP like the Medicines for Malaria Venture (MMV), disclosure of potential conflict of interest is an actual part of the employee's contract. Employees must sign conflict of interest declaration forms, which spell out perceived or potential, as well as real, sources of conflicts. With an organization like GAVI, as with many others, a conflict of interest arises when the personal or professional interests of an employee or board member, or indeed of its partners, are at odds with the best interests of the organization. In order to successfully pursue this policy, GAVI also applies specific COI policies to its different partners [9]. For example, representatives of GAVI and partners like the World Health Organization (WHO) and the United Nations Children Emergency Fund (UNICEF) are required to complete a COI disclosure form when executing contracts or participating in meetings where decisions for the procurement of drugs and vaccines are being taken [9]. For the Global Fund (GF), a COI arises when a "covered individual", that is someone who works directly with GF or is associated with GF, participates personally in taking decisions on any matter which affects GF and from which he or she might benefit. When this happens, GF expects all covered individuals to disclose the existence of any such conflict and the nature of such conflict. Secondly, all covered individuals must complete and submit a "Declaration of Interest" statement, which must be updated annually to GF's Ethics and Conflict of Interest Committee [10].

### Abstention and Withdrawal from Decision Making Process

The COI policies of several PPPs, operating within the field of agricultural biotechnology like the Maize and Wheat Improvement Centre (CIMMYT), the International Institute of Tropical Agriculture (IITA), World Agroforestry Centre (ICRAF), African Agricultural Technology (AATF), Convention on Biological Diversity (CBD), International Potato Centre (CIP) and Bioversity International (BI), which all receive funding from the Consultative Group on International Agricultural Research (CGIAR) state that apart from disclosing any conflicts of interest, individuals working for these organizations could elect to recuse themselves when decisions are taken on issues or organizations in which they have a secondary interest.

The IITA and CGIAR COI policies stipulate that board members are expected to formally declare their connection with any organization that would benefit from the organization's funding and withdraw from any decision that would be taken about matters concerning such a beneficiary [11,12].

### Training

Most of the PPPs that we surveyed require their employees to undergo periodic training on how to identify and avoid or manage COIs. The PATH COI policy talks about PATH's responsibility to train its staff to recognize conflict of interest situations as well as subject itself to internal and external monitoring to prevent conflicts of interest within the organization. The policy also indicates that the process of minimizing conflicts of interest must begin with the development of a mechanism to balance the conflicting interests of the parties involved in the partnership.

### Whistle Blowing, Public Reporting and Transparency

Beyond disclosure of a COI internally, some policies also featured public transparency of such COIs and ways to stimulate reporting of COI. For example, CIMMYT's COI guidelines emphasizes that a COI issue must be dealt with in an open and transparent manner that would not compromise the integrity and credibility of the organization [13]. Biodiversity International has a whistle blower policy, which actually encourages employees to secretly report instances of wrongdoing, which include COI by colleagues [14]. In the case of Global Fund, reports of COI occurrences are posted on its website.

### Independent Monitoring

Relatively few of the organizations use independent, external monitoring of COI, though there are some exceptions. The GAVI secretariat uses consultants who examine every potential COI with regards to procurement. The International Potato Centre uses a two tier external and internal review system to monitor all decisions and to prevent COI. The COI policies of several of the PPPs that we have reviewed rely on external monitors to assist in the prevention and detection of potential COIs. External monitoring is an effective way of continuing to reinforce trust among the partners of a multi-stakeholder organization; it helps to maintain an organization's ethical commitments. Though monitoring can be done internally or externally, external monitoring has the advantage of providing an independent and unbiased evaluation. An analogy here is financial auditing: an external financial audit offers an independent view on the finances of an organization and thereby serves to enhance the trustworthiness of the annual financial report. An organization that adopts a social auditing system of ethical and social monitoring and reporting can do so for a single domain (like conflict of interest). However, a more comprehensive, multifaceted approach to monitoring that involves all the activities that the organization is involved in might be more effective.

## Conclusion

Managing COIs is a critical governance and management function for PPPs. Based on our review of COI policies and the interviews that we conducted, a range of good practices was found. These included attention to accountability and governance, acknowledgement and disclosure of COIs, abstention and withdrawal from decision-making when a COI is present, reporting and transparency with respect to COIs, and independent monitoring.

One area that could perhaps use more focus, since it is at the frontier of the various approaches, is the use of external, independent monitoring. Though some PPPs have employed the services of external monitors to provide guidance and assistance on COI issues, these monitors are not usually fully cognizant of the activities of these PPPs, a situation which compromises the integrity of the external monitoring system. Most external monitors are called in to provide advice on conflicts after it has already occurred. Thus, in order to protect these PPPs, there may be a need to better manage COIs through integrating the services of 'an outside non-conflicted party' [15]. This outside non-conflicted party, (external monitor) could help to scrutinize, mitigate, and provide comprehensive guidance on ethical, social, cultural and commercialization issues by undertaking periodic assessments, while working alongside the team itself, by helping to assess policies and decisions and give objective advice throughout the partnership's progress. (Of course, the external monitor should not be doing other work for the organization that might seem to be dependent on the outcome of the monitoring.) We believe that this kind of system is a good way to help mitigate COIs within almost every area of activity in PPPs. In our work with an agriculture PPP, we conducted an external social audit [16].

It could be fruitful for PPPs to learn from each other on management of COI, and to move down the spectrum of policies and practices identified here towards independent monitoring of COIs. It might also be useful for this independent monitoring to extend beyond COI to other social objectives of the partnership.

## Competing interests

The authors declare that they have no competing interests.

## Authors' contributions

PAS and ASD conceptualized the idea and EBO drafted the first version of the paper. All authors participated in the data collection and revision for critical content. All authors have also read and approved the final manuscript.

## Pre-publication history

The pre-publication history for this paper can be accessed here:

http://www.biomedcentral.com/1472-698X/10/19/prepub
